# Experiences of eating difficulties in siblings of people with anorexia nervosa: a reflexive thematic analysis

**DOI:** 10.1186/s40337-022-00646-2

**Published:** 2022-08-20

**Authors:** Eleanor Scutt, Jasmin Langdon-Daly, Janet Smithson

**Affiliations:** 1 University of Exeter, Exeter, UK; 2 Somerset NHS Foundation Trust, Taunton, UK

**Keywords:** Anorexia nervosa, Siblings, Eating difficulties, Experience

## Abstract

**Background:**

Siblings of people with anorexia nervosa (AN) have been found to experience strong emotions, changing family roles and poorer wellbeing as a consequence of experiencing the effects of the illness on their sibling and family system. These factors, combined with genetic influences, may put siblings at an increased risk of developing eating disorder psychopathology in addition to other mental health issues. This research aims to explore the experiences of siblings of people with AN who have had eating difficulties themselves and investigate issues that may be important to the development and prevention of eating difficulties in this population.

**Methods:**

This qualitative study used a reflexive thematic analysis approach. Ten adults who had witnessed a sibling with AN and experienced eating difficulties themselves participated in semi-structured interviews.

**Analysis:**

Participants’ own eating difficulties were affected by the specific experience of witnessing a sibling with AN through mealtimes becoming emotionally charged, an increased focus on body size and diet, and comparisons with their sibling. Difficult experiences, such as marital discord amongst parents were common, as was a difficulty in managing emotions. The onset of AN within the family caused participants to take on caring responsibilities for their sibling and to hide their own difficulties for fear of adding additional burden to their parents. This reduced their perceived ability to access support and for some increased a desire to restrict as a coping mechanism for the stress they were experiencing. Systemic beliefs regarding the value of thinness were prevalent and influential. Protective factors, such as not wanting to become as unwell as a sibling with AN and an understanding of the negative consequences of AN, aided recovery.

**Conclusions:**

Eating difficulties in siblings of people with AN may be influenced by competition for slimness, increased focus on diet and body size, and a need to manage difficult emotions. The disruption to social connections and a difficulty finding emotional support that may be experienced by people when a sibling develops AN may further influence susceptibility to eating difficulties. Further research is needed into the best ways to support siblings of people with AN.

**Supplementary Information:**

The online version contains supplementary material available at 10.1186/s40337-022-00646-2.

## Plain English summary

This research investigates the experiences of people who have had a sibling with anorexia and had some eating difficulties themselves. Participants described difficult emotions after their sibling developed anorexia and many took on responsibility for helping their sibling. This was a stressful experience which some people managed by restricting their own eating. There was an increased focus on food and body size in the home, and this could also influence participants’ diet. Getting help could be difficult as participants did not want to burden their family with their own difficulties. This research concludes that when treating people with anorexia, the impact on siblings should be considered, and support should be offered to siblings to help them to manage the difficulties that this illness presents.

**Supplementary Information:**

The online version contains supplementary material available at 10.1186/s40337-022-00646-2.

## Background

Anorexia nervosa (AN) affects multiple facets of an individual’s functioning, including marked changes to mood, behaviour, and relationships [1]. Very low body weights associated with the condition cause energy deficiencies, impaired physical and mental abilities, and an increased risk of mortality [2]. Despite these impacts patients can show reluctance to change and concerned family members are often involved in compelling individuals into treatment and supervising eating up to six times per day [3]. The intensity of caring for someone with such an illness has been well documented [4–6], however, much of this research has focused on parents of people with AN with far less research exploring the effects on siblings. Siblings often have their lives disrupted due to changes in family relationships and the trauma of seeing a family member acutely unwell. Recent research has started to address this gap in the literature, finding that the wellbeing of siblings is negatively affected by experiencing a sibling with AN [7]. A meta-analysis across eating disorder classifications suggested that siblings may experience decreased quality of life, as well as increased isolation and psychopathology [8]. Such difficulties, coupled with an increased genetic susceptibility to AN in siblings, could put siblings at greater risk of eating disorder psychopathology.

Evidence surrounding the development of disordered eating in siblings of people with AN is mixed. Some research suggests that lifetime prevalence is around four times higher than the general population [9, 10]. Other research suggests no increased tendency to eating disorder pathology in siblings of people with eating disorders [11–13]. Qualitative studies report that siblings describe an increased awareness of food and a tendency to compare their bodies to their unwell sibling’s [14–16]. The impact of this on their eating habits was mixed, with some reports finding that they have developed a healthier attitude towards food [14, 15], some that it had no effect [11, 12, 17] and others suggesting that it was detrimental [16, 18].

Estimates regarding the heritability of AN vary from 48 to 74% [19, 20], suggesting a strong genetic contribution but not negating the impact of environmental factors. Environmental factors that increase susceptibility to AN may also be shared amongst siblings. Initial theories of the ‘psychosomatic family’ being influential in the development of AN [21] have been discredited with the suggestion that any disturbances in family functioning may be the result of AN rather than a causal factor [22]. However, elements of the family or cultural environment shared by siblings may be influential in the development of eating disorders. Research suggests that restrictive diets and negative talk amongst mothers about their own or others’ body image had a detrimental effect on daughters’ eating behaviours, including the prediction of restrictive eating behaviours [23–25]. Family scripts around healthy eating or the need to be thin may be shared amongst siblings [26], and could contribute to a preoccupation with body image and a desire to alter eating patterns to control this. Culturally bound societal messages about the need to be thin may also contribute to this, particularly in Western societies [27].

Research suggests that psychopathologies such as depression and PTSD show symptom transmission amongst families, with non-genetically related relatives experiencing similar symptoms [28–30]. This contagion effect may also be present in eating disorders with suggestion that parents may project a desire to be thin onto their children or model unhealthy eating behaviours [31, 32]. Additionally, where parents have an eating disorder, this could influence the ability to parent effectively and may be associated with discordant marital and family relationships that in turn increase the risk of eating disorder pathology [31]. Contagion effects of AN have also been reported amongst peers [33, 34], with studies finding that peer’s body mass is negatively associated with the likelihood of an individual having AN [35]. However, little research has investigated symptom transmission or contagion between siblings. Given the competitive and comparative nature of AN and the fact that siblings are frequently in the high-risk age groups simultaneously, it is surprising that this has not been considered.

The cognitive interpersonal maintenance model of AN suggests several factors are relevant to the development and maintenance of the illness, including thinking styles, social and emotional factors, the valued nature of the condition and the reactions of others [36–38]. Trauma is a possible predisposing factor that may be shared among siblings: approximately two thirds of people diagnosed with AN report having experienced a major life event or trauma in the year leading up to the illness [39]. Relational problems within the family or with friends are commonly cited traumatic events that may equally affect a sibling. Alternatively, the traumatic event of witnessing a sibling develop AN may constitute a non-shared factor that precedes the development of eating disorders in siblings.

Evidence suggests that rigid- and detail-focused thinking styles are common in people with AN and may be both predisposing and maintaining factors of the illness [36]. These features may have genetic links, with well siblings of people with AN exhibiting weak central coherence [40–42] and showing similar deficits in set-shifting [41, 43]. A rigidity of thinking patterns amongst parents may make them more likely to respond to their child in a way that is controlling or critical [44], therefore creating a stressful home environment for the whole family. Alternatively, parents may become overly permissive in an effort to reduce tension in the home, potentially enabling the continuation of disordered eating. Either of these responses may unwittingly maintain the illness [36, 45], and may increase sibling’s worry or cause them to take on a new role in the family. Many siblings of people with AN report their roles in the family changing following the development of the illness: becoming carers themselves, mediators in family disputes or becoming distanced from the family [8]. The combined difficulties of changing family roles, coupled with feelings of fear, guilt and sadness about their sibling’s illness [16, 46–48], make this experience very distressing. Additionally, well siblings may struggle to discuss their feelings due to a reduced availability of family members and a desire not to burden them further [49, 50]. These difficulties are discussed in the model as social and emotional maintaining factors.

Despite evidence of the impact of AN on siblings and the possible increased risk of eating disorder pathology, support for siblings is not routinely offered. Although they may be included in family therapy, the dominant treatment for adolescents with AN, this does not focus on their individual needs, indeed siblings may be tasked with taking on a support role as a part of this treatment. Siblings may be reluctant to discuss their own difficulties for fear of adding extra burden to their family [8]. Research is needed to investigate where to best target support and what forms of support would be beneficial. By exploring the experiences of people who have both witnessed a sibling with AN and had restricted eating themselves, this research aims to investigate what relevant factors led them to develop a similar illness, particularly considering the potential contagion of AN. We aimed to answer the following research questions:

- How did having a sibling with AN influence participants’ own eating difficulties?
- What factors do participants think were relevant to the development of eating disorder symptomology in themselves and their siblings?
- What were participants’ experiences of support following their sibling’s difficulties?

## Method

### Design

A reflexive thematic analysis methodology [51] was used for this study, allowing for identification of themes across the data set that could give specific ideas for systemic change.

### Data collection

The first author developed a semi-structured interview guide based on the principles of the cognitive interpersonal maintenance model of AN with additional questions around the development of eating difficulties. It focused on the following areas: broad experiences of eating difficulties in interviewees and their siblings, relevant factors in the development of disordered eating, management of emotion and what support would have been useful (see Additional file 1). Proposed questions were reviewed by an individual with lived experience from the eating disorder support group at the University of Exeter. Following this review, the interview questions were amended to increase their focus on key issues and relevance to potential participants. A pilot interview was conducted with an individual with experience of a sibling with AN but no eating difficulties herself. The interview schedule was then revised to reduce the number of questions and make them more targeted to the research questions.

### Ethics

This study received ethical approval from the University of Exeter ethics committee (approval number: eCLESPsy001999). Participants were given an information sheet and returned a signed consent form prior to participating. Due to the sensitivity of the topic, participants were reminded throughout the interview of their right to stop at any time or to skip questions. At the end of the interview, the first author checked on the participants wellbeing and offered a debrief. All participants have been given a pseudonym and identifying details have been omitted from transcripts.

Participants were given details of the study’s social media pages to view the published version of this study. The authors have taken care to be respectful and non-judgemental in representing the views and experiences of participants.

### Participants

Purposive sampling was used to find participants who identified as having developed restrictive eating difficulties following experiencing a sibling with AN. Restrictive eating difficulties were defined as limiting food intake (with or without increased exercise and purging), with a desire to alter their body shape or weight for a period of at least 3 months, resulting in them rapidly losing weight or becoming underweight. Participants were required to be over the age of 16, to have been at a healthy and stable weight for a minimum of three months and not currently experiencing mental health problems.

Recruitment was done through online adverts on social media sites: Twitter, Instagram and Facebook. Paid-for adverts on Facebook and Instagram were targeted at people who followed prominent eating disorder accounts, such as national eating disorder charities, and those who were interested in diet, nutrition or mental health subjects. Recruitment was worldwide but targeted at English speaking countries to maximise the chances of finding eligible participants. Adverts were also posted on research platforms—MQ: Participate and Call for Participants—and on two charity websites in the UK and in the USA.

Participants who registered interest and were assessed as eligible were invited to a Zoom interview. Eleven participants (nine women and two men) completed interviews lasting 40–50 min, one man was subsequently deemed ineligible due to not meeting the eating issues criteria. One participant chose a telephone interview.

### Data analysis strategy

The first author conducted a reflexive thematic analysis guided by the principles set out by Braun and Clarke [51–53]. An interpretivist paradigm was employed, focusing on how the meanings made by participants of their experiences may be influenced by the author’s own experiences and knowledge [53]. Both inductive and deductive approaches were used [51], such that theory was used to guide interview questions and influenced the author’s perspectives of how to interpret the data, but open coding was used to understand the meanings made by participants rather than fitting these to a theory. Cross-checking of part of one transcript by three other researchers allowed for a meaningful discussion around different perspectives of the participant’s experience, and helped the first author to consider the impact of their own knowledge and assumptions on their interpretation.

The analysis was guided by the six steps for thematic analysis outlined by Braun and Clarke [51, 52]. This was an iterative process; the first author moved between phases, regularly returning to the original transcripts to ensure that emerging themes were grounded in the data.

For each sub-theme the first author identified a list of supportive quotes, quotes were chosen for the write-up based on their relevance to the theme and significance to the participant’s story. Member checking of synthesised analysed data was offered to all participants and completed by three.

### Reflexivity

In approaching this research, I (the first author) was aware that my position as a white, female, trainee clinical psychologist would influence my questioning during the interviews and my interpretation of the data. Throughout the interview process I kept a reflexive diary to record my feelings and assumptions, and how participants’ accounts fitted with my prior knowledge [54]. To increase validity, decisions made during the analysis and rationales for these were documented [55].

## Results

Participants were aged between 21 and 33 years of age (mean = 26.7), all identified themselves as White. Seven were resident in the UK, with the remaining three from New Zealand, South Africa and Ireland. Two participants had received a formal diagnosis of AN, eight had received no eating disorder diagnosis. Four reported previous episodes of depression, one had previously received a bipolar type 2 diagnoses and one an obsessive–compulsive disorder diagnosis.

All participants reported having a sister with AN; in one case this was undiagnosed and in a further case the participant was unsure of formal diagnosis (see Table 1). Participants had between one and six full siblings (mean = 2.1). Nine participants developed eating difficulties after their sibling’s diagnosis, one developed AN before their sibling but relapsed when their sibling was diagnosed.

**Table 1:** Demographic information for each participant

	Nature of eating difficulties	Sibling’s diagnosis	Participant’s age when sibling developed AN	Birth order (number of full siblings)	Birth order of reference sibling	Engagement in family therapy
Heather	Undiagnosed restriction of eating plus overexercising	AN	16	1 (1)	2	No
Andrea	Undiagnosed restriction of eating	AN presentation- unsure if diagnosed	10	2 (1)	1	No
Sarah	Undiagnosed restriction of eating plus bingeing and purging	AN	14	2 (1)	1	No
Bobbi	Undiagnosed restriction of eating, binge eating and purging	AN	16	3 (3)	4	No
Sophie	Undiagnosed restriction of eating and purging	AN	16	1 (2)	2	Yes
Chloe	Diagnosed with AN and Bulimia Nervosa	AN presentation- undiagnosed	Late 20’s^a^	2 (1)	1	No
Rachel	Diagnosed with AN	AN	12	1^b^ (2)	1^b^	Yes
Holly	Undiagnosed restriction of eating plus overexercising	AN	16	1 (1)	2	Yes
Hannah	Undiagnosed restriction of eating and purging	AN	15	1 (6)	6	No
Paul	Undiagnosed restriction of eating and overexercising	AN	21	2 (2)	3	No

^a^Participant developed an eating disorder before her sister but relapsed after her sister’s illness

^b^Participant was a twin

The themes and sub-themes developed from the data are shown in Table 2.

**Table 2:** Superordinate and subordinate themes developed during the analysis

Superordinate theme	Subordinate theme
Eating difficulties were influenced by sibling’s AN	Emotionally charged mealtimes at home Comparison and competition Increased focus on body image and diet
Changing eating patterns to manage difficult emotions	Feeling responsible for a sibling with AN Restricting eating to manage emotions Traumatic experiences increasing the need for control
Systemic pressure to be thin	Family beliefs about diet and body size The thin ideal
Finding appropriate support was difficult for participants	Being the well sibling Difficulties getting support Types of support that may have helped

### Eating difficulties were influenced by sibling’s AN

#### Emotionally charged mealtimes at home

Mealtime supervision, a key part of treatment for AN, is often very difficult for people with AN who feel fearful and stressed at the prospect of having to eat, and for their families who are tasked with enforcing meal plans. Six participants spoke of how this caused them to associate mealtimes with stress.

Sophie: “There was a lot of stress a lot of crying and shouting and throwing food and things, which doesn't really help when you have your own digestive issues.”

Participants also discussed the difficulties of having to follow controlled eating plans and show a good example.

Holly: “I felt sort of trapped by it, it was always like ‘you have to eat enough to show her a good example, you have to’.”

Losing control of their eating patterns and diet appeared to be a trigger for some participants to restrict their eating as a way of feeling in control as soon as they could. Four participants spoke of restricting their eating once they left home and were no longer being pressured to eat.

Sophie: “When I went away to university I just kind of saw it as like an opportunity that like they can't worry about me now, I’m not at home anymore so I saw it as free rein to sort of restrict a lot more dramatically.”

In contrast, Andrea stopped restricting her diet when at university as she learnt more healthy patterns of eating from those around her.

Andrea: “I think it was just kind of seeing people having like three meals a day and like having snacks in between and not worrying about like the fat content and certain foods.”

#### Comparison and competition

Body shape comparisons were discussed by all female participants regardless of whether their unwell sibling was older or younger. This appeared to be particularly important for participants who had one female sibling.

Sophie: “She was 13 at the first diagnosis and I was 16 so it wouldn't have made sense any way to compare our bodies, but I definitely did, and I definitely felt very jealous of her in a sick sense.”

Sophie’s account suggests an awareness that wanting to be as thin as her sister was unhealthy but that there was a part of her that still aspired to be that way. For Andrea, this comparison was an imagined sense that others, including her sister, were expecting her to look a certain way, underpinned by a societal norm of thinness as an aspiration.

Andrea: “The feelings of just like she's older than me like, quite substantially older than me and she's wearing smaller dress sizes than I am, like what does what must she think of me, or what does that mean about me.”

This appears to convey a sense that in her role as ‘younger sister’ Andrea feels she should be smaller, and perhaps should be the one to be looked after. Within this narrative her sister becoming unwell has given Andrea a sense of unease at the disruption of family roles.

A desire for thinness was not the only basis for comparison; Holly spoke about restricting her eating to appear strong and perfectly healthy rather than thin, but also spoke of feeling competitive with her sister.

Holly: “I’m naturally quite competitive as well, so as soon as her eating disorder started comparing then I’d naturally compare back you know, want to be better.”

This sense of competition could lead to a cycle of siblings triggering one another to restrict their eating. This was described by Chloe who developed AN first and struggled whenever she noticed her sister becoming thinner.

Chloe: “We went shopping and she was tiny, and I remember that made me feel rubbish again and I started to restrict my eating, I don't know what it is, is it a fear of she's going to be thinner than me?”.

In some cases, comparisons between siblings were explicit. Rachel described her sister becoming violent towards her out of jealousy when she became thinner.

Rachel: “She'd had to gain weight and she was a healthy weight, and I went in quite underweight which she didn’t like so she used to beat me up.”

Rachel did not have a desire to be thinner than her sister but stated that experiences like this brought up difficult emotions that perpetuated her mental health struggles and restrictive eating.

#### Increased focus on body image and diet

Even for those who did not directly compare their bodies to their sibling’s, the increased focus in the home on diet, exercise and body image could be contagious.

Paul: “The constant conversations about food have definitely made me think about food and I’d mirror that behaviour and check what’s in food and obsess a bit more.”

Paul did not have eating difficulties before his sister became unwell but had experienced anxiety; an increased awareness of food became something for him to worry about and want to have control over.

The focus on diet and weight within the home when someone has AN can be inescapable and for participants this could make recovery difficult.

Rachel: “In my recovery I haven't been able to forget about having an eating disorder because my sister speaks to me every day about it, and I have to visit her every week when she weighs five stone and has a tube up her nose so it's just really difficult to ever move on from.”

Rachel discussed the need to have space from her sister and to prepare mentally for visiting her as she is aware that visits can be a challenge in her own personal recovery.

### Changing eating patterns to manage difficult emotions

#### Feeling responsible for a sibling with AN

Participants took on responsibility and guilt for their sisters eating disorder for a range of reasons including not noticing it early enough, thinking that they caused it and being unable to save them.

Sophie: “I became like health obsessed when I was a teenager and I sort of shared those things with my sister, so I always felt like I put her on the path to her eating disorder.”

Sophie remembers feeling guilty as a child that she may have influenced her sister’s eating disorder. This may have increased the sense of responsibility that Sophie felt towards her sister and her family.

Sophie: “I wouldn't say I parented as such, but I definitely felt very responsible, felt very guilty, I felt like it was my job to help and stuff which you know I didn't mind helping, but I do think the stress again played a big part in later mental health issues.”

Andrea’s sense of responsibility came from a guilt at not realising that her sister was unwell at first.

Andrea: “I've felt like I could be the one to like save her, like if I was as thin as she was or just maybe not as thin as her, but maybe like an acceptable level of thinness to her and she saw me eating, then she might think it wasn't so bad to eat.”

For Rachel and Andrea this assumed responsibility resulted in failed attempts to help their sibling, bringing about complex emotions.

Rachel: “I just had too much hatred to myself, I think I blame myself for (sister) as well. I told myself if anyone can get her better I will, so I think mine was more a hatred towards myself.”

Andrea: “I think at the start I was kind of like her champion, ‘I'm there for you, I will do anything for you’, and it was after a few years of her consistently lying to me about it all that it just I just felt quite betrayed.”

Over several years of witnessing a sibling with an eating disorder, through cycles of relapse and remission, emotions and attitudes towards a sibling may change. Andrea explained how after years of trying to help her sister she now feels upset that, despite her efforts, her sister has been unable to recover and betrayed that the illness has caused what she perceives as consistent lying from her sister. Her sense of anger and betrayal is compounded by her family’s reaction to always put her sister’s needs first, in this context Andrea perceives her parents as complicit in her suffering by siding with her sister. In contrast, Hannah felt that her attempts did help her sister recover, however, this placed a high responsibility on her to help.

Hannah: “I think it actually brought us closer together because she sort of, not listened to me, but I could reason with her more than what my mum could.”

#### Restricting eating to manage emotions

All participants described having strong emotions towards their sibling when they were unwell with AN. For some, the intensity of emotions and difficulty knowing how to manage them led to increased anxiety, depression and restrictive eating patterns as a method of coping.

Sarah: “I think a lot of it would just be in times of stress when I felt like I needed to control something.”

Stress was often borne out of fear and uncertainty of what might happen. Many participants feared for their siblings’ lives when they were unwell and struggled to know how to cope with this feeling.

Bobbi: “I found it incredibly scary at the time, it felt like an overload of fear all the time that something was going to happen long-term and that she was essentially just killing herself slowly.”

For many participants emotions were complex, changing and difficult to discuss.

Sophie: “I definitely think that anger is a difficult emotion to process, especially because I can feel very guilty over anger, like the anger that I felt towards my sister, I still feel very, very guilty over that and I haven't forgiven myself for the way that I treated her.”

Sophie’s parents were in the process of splitting up at the time of her sister’s illness and her anger resulted from many different things including uncertainty and insecurity in her family relationships, worry for her sister and a difficulty understanding her sister’s struggles with the illness. In her anger Sophie is standing up for her own needs but also perhaps showing her disappointment that her hopes for her sister’s recovery had not been realised. A subsequent better understanding of the illness has helped to reduce her anger but also has increased her retrospective guilt over this. Sophie’s guilt around her anger and previous treatment of her sister is testament to this behaviour being unaligned with the values she holds for herself as a supportive sister.

Although participants described restricting their eating to feel more in control and to manage emotions, they were also aware that this compounded the guilt they felt for acting in a similar way.

Paul: “I am such a hypocrite because I’m just endorsing this behaviour but then on the other hand trying to stop this behaviour.”

Paul described how the difficulty in managing his own eating difficulties whilst supporting his sister was compounded by guilt and fear of modelling unhealthy eating behaviours.

#### Traumatic experiences and the increasing need for control

Bobbi and Rachel spoke about emotional and physical abuse from their parents which they thought may have influenced their troubled relationship with eating and their bodies. Both participants said that their weight had been one of the things that they felt criticised for.

Rachel: “I think my mum picked on both of us for our weight.”

Bobbi: “I think it taught me a lot of self-hate. I think I got it into my head that I didn’t look the way I was supposed to, so I wasn’t good enough.”

For Bobbi this directly affected how she saw her body and influenced her desire to lose weight. Rachel experienced several other difficulties such as her twin becoming severely unwell and a break-up of her family. She described how all her family members struggled to cope at this time and she had nobody to turn to for support. For her having an eating disorder gave her something else to focus on.

Rachel: “I suppose, having other problems kind of took a lot of it away.”

For both Bobbi and Rachel their initial difficulties with eating therefore became attempted solutions to manage intolerable pressures at home.

No other participants mentioned any forms of abuse. However, six mentioned other difficult childhood experiences, such as marital discord and divorce in parents shortly before or after the development of an eating disorder. Participants cited this as a factor in triggering difficult emotions and wanting a sense of control that could be achieved through restricting their eating.

Chloe: “I think the trigger was when I turned 14 my mum had an affair, and I think the shock of it was just you know really devastating as a teenager and I think my immediate reaction was, you know that's something I can control like I’m going to stop eating.”

Chloe’s eating difficulties also started as an attempt to divert her emotions elsewhere following distress within the family system.

Experiences of health issues either for participants or for their parents were also mentioned as factors increasing emotional distress and a wish to have more control.

Heather: “My dad had a heart attack when I was about nine and my sister was six, I remember my mum fully going on a health kick then… my mum was like we need to be healthier as a family.”

For Heather this increased the focus on her diet and was an early reinforcement to healthy eating in the context of her father having a heart attack.

### Systemic pressure to be thin

#### Family beliefs about diet and body size

Family scripts around a need to be thin and modelling from parents around eating was discussed by many participants as a possible antecedent to their eating difficulties. These included observations of parent’s approaches to their own diets.

Heather: “Mum does that thing, where she’ll also punish herself, she’ll be like ‘Oh well, I shouldn’t have that piece of cake, because I didn't go for walks today.”

There were also accounts of parents giving explicit instructions to their children to be thin.

Bobbi: “(My father) would very often restrict what we ate and how much of it we were allowed to eat, and there were a lot of different rules for everybody in our house because we looked different.”

Her father’s restriction of what she and her sisters could eat, in the context of abuse, was perceived by Bobbi as a causative factor in all her sisters developing eating disorders. On the other hand, the encouragement by her mother to restrict her eating was seen by Sophie to be underpinned by normative assumptions about dieting, the impact of which she perceived that her mother was unaware of at the time and later regretted.

Sophie: “(My mother) sort of said to me ‘Oh, you know you can try this diet that I tried when I was your age and that'll help’ and obviously she regrets that now, but at the time she just thought that was normal, like teenagers go on diets.”

This was not consistent across all participants; two participants recalled no pressure from parents to engage in restrictive weight loss practices.

Holly: “We’d never been restrictive at all at home, and I’d never seen my parents diet or anything like that.”

#### The thin ideal

Messages around the thin ideal were seen to be widespread in the media, schools and in general society. Viewpoints on thinness were often dissonant, with participants acknowledging that AN was an awful illness that they would not wish on anyone, whilst also holding aspirations towards thinness. Nine participants were aware of this contradiction and spoke of the difficulty of wanting to manage their weight but not become unwell.

Hannah: “I didn’t like what I’d seen on her, I don’t think she looked good, she looked ill, I remember thinking I would never want that to happen to me sort of thing. But kind of very similar did.”

There were different ways of managing this dissonance: Sophie spoke of a previous belief that she could diet without it becoming unhealthy for her but has since learnt that this was not possible.

Sophie: “I always felt like you should straddle the line between being just you know just thin enough and go no thinner than that, and that was what I was like my sister is gone too much that way she let it get out of control, but I have it in control, I can just do it just enough.”

Other participants managed dissonance by distancing themselves from their sisters’ illness and minimising their difficulties.

Sarah: “You don't have anorexia because you know what that looks like so what are you doing, like feeling like you're kind of making a fuss.”

This initially increased Sarah’s wish to restrict her eating but this passed over time.

Sarah: “There were periods where I would have like imposter syndrome and I would be like well I’ve just got to like double down on this and I’ve gotta really restrict because then it will justify what I’m doing. But then, on the other side of that, probably towards the end of the periods, I would be like well I just need to stop, because this is ridiculous and I’m better than this”.

An awareness of the possible dangers of trying to be thin and a worry about becoming as unwell as their sibling was protective in preventing further eating difficulties.

Heather: “I think I worry that if I was to get into the same habit as my sister like I’m very conscious of it, like I don't calorie count now, I don't weigh myself, I don't do any of that because I almost worry that I would end up like her.”

All participants noted that managing the internal struggle between the desire to be thin and yet to avoid becoming unwell was a challenge and that witnessing the struggles of a sibling with AN could both motivate recovery and risk relapse.

### Finding appropriate support was difficult for participants

#### Being the well sibling

Participants were acutely aware of the stress that their sibling’s eating disorder had on their parents and described putting pressure on themselves to eat well and appear emotionally healthy.

Heather: “If I don't have seconds at meals (my mum) will make comments like ‘Why? What's wrong? Why are you not having seconds? Are you unwell?’”.

Eating could become a performative act to parents and siblings to demonstrate ‘being well’ and this caused participants increased stress. Heather now has a mostly healthy eating pattern but still feels a need to restrict her eating before and after a visit home to compensate for having to eat more than she usually would when there.

Heather: “I was always very conscious that when I would go home I’d be eating loads and so I’d have to be careful the week after, in my head I’m always like ‘you're going home, you're going to be eating loads make sure you don't eat too much in the week after or week before’.”

Playing the part of the well sibling was a response to an awareness that the family system was already stretched, and participants wished to hide their difficulties to prevent it from becoming overwhelmed.

Heather: “I thought, they’ve got one unwell child they don’t need another one, so I’ve just never really spoken to them about it.”

Sarah also found it difficult to talk to her parents about her own experiences, despite them trying to discuss it with her. She attributed this to not wanting to burden her parents further and not wanting to put extra guilt on her sister.

Sarah: “Everyone's like rallying around to look after this person and you feel like you kind of have to be the mature one and, like not get into much trouble and just make sure that you're looking after things because you don't want to put extra stress on what is already going on.”

Sarah also recognised that she has difficulties understanding and expressing her emotions and her eating difficulties developed partly in response to this.

Sarah: “It's like you don't know how to open up to these people around you so here's something that you can do to control everything that's overwhelming.”

Four participants discussed a desire to hide their emotions from their families and restricting an eating as a way to help them cope. Problems with processing emotions may reflect an internalised desire to be the well sibling and an unwillingness amongst participants to allow themselves to experience these for fear of increasing the burden on their family. This may also be dependent on the family culture and context and how this is internalised.

Sophie: “I wouldn't have felt like I could say you know that it's a bit too much or whatever, because I did grow up in a Christian household as well, so it was very much like, you know, you should self-sacrifice.”

Sophie discussed how her Christian values meant that it was difficult for her to accept feelings of being overwhelmed.

Parents supporting one child with AN may lack time or emotional availability to support their other children. Andrea described never feeling that her parents cared for or prioritised her needs, leading her to feel upset and angry.

Andrea: “They always put her needs like in front of mine at my expense, like not just even like when it doesn't affect me, like when things you know would kind of harm me, it would be her needs first”.

Andrea’s perspective on this has changed over time, initially she felt happy to surrender her needs but over time, as her sister has experienced multiple relapses, Andrea has become more vocal in expressing her own needs and wish for support. However, other participants stated that their siblings’ illness brought them closer together as a family.

Sophie: “I think I actually became more dependent on my mum because of the whole thing and possibly her on me as well a little bit like we became close.”

Sophie spoke of an awareness that her mum was feeling stressed and overwhelmed. Although Sophie did not feel that she took on a parental role, the need to offer emotional support to her mum meant that family roles became less defined. This may have affected her ability to stay in her role as a child and a sister. Although participants expressed a desire to support their family, maintaining the role of a sibling was difficult for participants who were keenly aware of their parents feeling overwhelmed.

Heather: “It was just me and her and I couldn't really leave her in the house alone, so I was just trapped for a month with her the only time I went out was either to walk the dog or go to the shops it was just a bit of a hermit life.”

Heather felt under pressure to allow her parents a break and therefore took on caring responsibilities for her sister whilst revising for exams. She spoke of taking on the role of carer whilst also being a student as being draining and isolating.

Paul took on a role as a carer and advocate for his sister due to a feeling that his family did not take her illness seriously. This has had a detrimental impact on his relationship with his sister and with his family.

Paul: “I am the one person that she hates, because I am the one person who tries to interfere with the eating disorder and help. My mum says ‘just be her brother’ and I’m like, ‘well I could be her brother if you were helping her, and then I could stay out of it’.”

Paul has struggled to communicate his own needs to his family as he has prioritised his sister’s needs and worries that he will be accused of hypocrisy if he discussed his own issues with eating. Both Paul and Heather have managed the stress of taking on a parental role by moving far away from their families to ensure that their caring responsibilities are time limited.

For some participants, siblings provided a source of support, particularly when both had recovered from similar illnesses as this fostered a sense of shared understanding.

Bobbi: “We confided in each other a lot, we found a lot of support and love with each other.”

Bobbi described becoming very close to her siblings as a way of supporting one another with eating difficulties and with their shared experiences of abuse, offering one another support where this was lacking from their caregivers.

#### Difficulties getting support

Possible barriers to seeking support are numerous and six participants alluded to worries about being stigmatised, being unsure how to seek help or their needs not being great enough for professional intervention. Andrea said that she struggled to ask for support because her parents were busy, and she was worried about being assumed crazy or being put in hospital.

Andrea: “I just thought I’d be in trouble I didn’t know what was okay, and what wasn't okay, in terms of the mental health spectrum.”

Andrea’s experience suggests that she found it difficult to know what a normal level of distress was, perhaps this was skewed by comparison to her extremely unwell sister.

No participants in this study were offered individual support from a professional because of their sibling’s illness. Most participants who had sought support pursued this themselves and either had private therapy or six sessions of individual therapy. Three interviewees participated in their sibling’s family therapy but found that this did not help in supporting them and could add to the difficulty of their sibling’s illness.

Sophie: “I would have liked it if I’d had one-on-one, but they didn't offer that, it was just as the whole group, and I think my sister found it really embarrassing as well, and I remember the counsellor was asking me about how I felt about my parent’s divorce in front of my parents. I remember thinking ‘How is this helpful for me?’.”

Sophie occasionally participated in her sister’s family therapy sessions, this quote highlights how these may have increased her feelings of distress as well as bringing the emotions of other family members into focus. This was a difficult experience for Sophie and the lack of individual support or consideration of her needs may have had an impact on her wellbeing.

#### Types of support that may have helped

There was a desire amongst all participants for more support when their sibling became unwell. They suggested that individual support, education in schools and support groups might have helped them to understand and manage their emotions around the situation at home.

Andrea: “Just having someone to say’It's okay if you're feeling xyz, it's normal to feel xyz even though it's like a scary feeling to have but it's normal and that's like in the normal experience, how can we help support you through that?’”.

Support for parents to help them cope better with difficulties was also suggested as potentially being beneficial and may allow them to better support other siblings in the house.

Holly: “If my parents had some of the load taken off them, I feel like I don't know if my problems were serious enough to need serious psychological help, it was more I just needed the support of my parents.”

Holly’s sister had experienced several hospital admissions and had engaged with family therapy and meal supports in the community. This had put a lot of responsibility on her parents to support their unwell child and as a consequence they were less able to focus on Holly’s needs.

Holly: “I think the main thing was, that helped was just distancing in me being away at Uni and then that kind of allowed (sister) to shut me off, as part of the disorder and resolved all those issues.”

In the absence of this support Holly found that the only solution to improve her wellbeing was to distance herself from the family system. This need for space from a difficult family environment was echoed by six other participants including two who had received intensive treatments themselves. This suggests that the chances of recovery from eating difficulties may in some cases be facilitated by increasing respite from the daily challenges of living with a sibling with AN.

## Discussion

This research aimed to investigate the perspectives of siblings of people with AN on factors they deemed to be relevant to the development of their own eating difficulties and how they navigated their sibling’s illness and their own recovery. Having a sibling with AN had a significant impact on the lives of all participants, most of whom were adolescents when their sister’s illness began. Feelings of responsibility for their sibling, changed roles within the family and a need to present as healthy were consequences of their sibling becoming unwell and impacted on participants’ wellbeing. In addition, traumatic experiences and a want to gain control of difficult and uncertain situations were a catalyst for eating difficulties for some participants. Direct effects of witnessing a sibling’s illness and treatment, such as an increased focus on food and body weight, comparisons between siblings and having to role-model eating were all pertinent factors in participants’ own struggles with eating. Societal idealisation of thinness and difficulties in accessing appropriate support presented barriers for participants in getting well.

Consistent with some previous findings, participants in this research described their body image being influenced by their sister’s appearance [16]; this effect was stronger for younger siblings [56]. In this study, participants appeared particularly susceptible to compare their bodies with one another if they were both sisters with no other siblings that were close in age. Birth order appeared to be relevant to some participants in their changing roles following their sibling’s illness, particularly for participants who were younger than their sibling with AN. Comparisons either encouraged restrictive eating or motivated participants to eat to avoid becoming as unwell as their sister. Several participants held both views concurrently and discussed a previous desire to find a perfect level of thinness. These aspirations were underpinned by systemic messages around thinness being desirable, notably all participants were White and came from Western cultures where slimness is idealised. An idealisation of thinness was pervasive amongst several of the participants’ families, with all but two talking about their parents’ own restriction of certain foods, encouragement of compensatory eating behaviours or general negative attitudes towards fatness. This may evidence a contagion effect in families where parental beliefs about the value of thinness and an expressed desire for their children to be slim translated into restrictive eating patterns in participants and their siblings [31]. For participants in this study, all of whom had recovered, this had at some point been outweighed by an understanding of the risks of the illness and the detrimental impact it has on lives.

The cognitive interpersonal maintenance model states that poor social connections may be linked with the onset and maintenance of AN [36–38]. Interviewees described changed family relationships following the onset of AN in a sibling and for many this resulted in increased distance from their family. Participants described being acutely aware of the strain that their parents were under and wanting to hide their own struggles for fear of increasing the burden on the family. This led to participants playing the part of being well, potentially increasing feelings of isolation and emotional distress as parents were unaware of their struggles. Furthermore, several participants spoke of traumatic events in the family that immediately preceded the development of AN within the family and led to strained relationships. This may have increased susceptibility to eating difficulties in participants. Reconnecting with family whilst a sibling still has AN may make recovery difficult; some participants only felt able to recover when their sibling recovered or when they were able to distance themselves from their family and make connections with others.

Feelings of sadness, fear and responsibility have been well documented in well siblings of people with AN [8, 15, 47, 48, 57]. Consistent with the cognitive interpersonal maintenance model, some participants said that their eating difficulties arose as a consequence of the stress of managing intense emotions following their sibling’s illness. This link between stress and eating difficulties is consistent with other research [58–60]. Sources of stress included: relational tensions within the family, major life changes and taking on responsibility for their sibling’s health. Several participants found it difficult to discuss their emotions due to not wanting to burden parents, parental availability, and shame around the content of these emotions. Minimisation of their difficulties and feeling that these were not sufficiently bad to seek support were also barriers for participants in getting help. However, some participants noted that they became closer to their family following their sibling’s illness and that this has helped them in recovering from their eating difficulties. Establishing connections with others is an important part of treatment for an eating disorder [37] and finding ways to help families build their relationships may be important to improving the wellbeing of the whole family.

### Strengths and limitations

A strength of this study is that it presents an in-depth exploration of the perspectives of siblings of people with AN, an under-researched population in this field, on their experiences of eating difficulties. The integration of multiple perspectives and experiences in this research is beneficial in that it has allowed me (the first author) to develop overarching themes, combining my interpretation of participants’ stories and previous research. The reliability and validity of the coding and interpretation of the data is enhanced by the use of member checking of synthesised analysed data and cross referencing with other researchers, however, this may have been further improved through the use of member checking on the coding of individual transcripts [61].

The population was relatively homogenous in that all participants came from Western cultures and identified as White and cis-gendered. The scope of this research is that it primarily focuses on female experiences, only one participant was male. All spoke in reference to a sister with AN, this gender focus is common with much research in the field of AN [62].

### Reflexivity

As a White female with no history of an eating disorder and limited experience of working with people with eating disorders, I (the first author) am aware that many of my preconceived ideas regarding eating disorders were based in my reading and in cultural narratives around the illness. For example, in my reflexive journal I noted that I held an assumption that all participants would subscribe to an idealisation of thinness and that seeing a sister become thinner would bring about some ideas of desirability of the illness for participants. Whilst the thin ideal was present in my interpretation of the data, I also recognised contradictions to this and that for many emaciation was not seen as aspirational.

Throughout this process, it was difficult to separate my dual roles of researcher and clinician; this was particularly difficult when speaking to participants who had had negative experiences of the health system. In these instances, I found myself becoming critical of the system and wanting to support participants rather than engaging with the content and meaning that interviewees were providing. By focusing on my role as a researcher and the purpose of the research I was able to manage this struggle and limit the impact on the individual interviews.

Staying engaged with participants’ experiences both during interviews and analysis was also challenged by the dominant positivist narrative present in clinical psychology [63]. This approach suggests an objective and measurable truth that, whilst useful in developing concrete evidence-based treatments, may ignore the complexities and contextual factors that determine individual experiences [63]. At times I felt myself being steered towards positivist narratives to develop generalisable implications. This was influenced by a sense of duty to participants to maximise the possible impact of their stories, however, this approach would have done these participants a disservice by ignoring the complexities and nuances of their experiences.

### Clinical implications

Consistent with the findings of previous research [8, 16], siblings of people with AN wanted individualised support when their sibling became unwell. As a potentially high-risk group, individual support should be routinely considered as a preventative measure to help manage the stresses of living with someone with an eating disorder and attend to other risk factors. Multiple sources of information about eating disorders from mental health services and schools would also help in allowing siblings to understand AN and to prevent eating disorder psychopathology.

Advice for parents on how best to support siblings through not pressuring them to eat and avoiding comparisons between siblings should be incorporated into treatment for AN. Family therapy should address the potentially changed roles within the family and help to ensure that siblings are not taking on parental roles or assuming responsibility for their unwell sibling’s health. Work around family scripts and beliefs about body size and diet may also be useful in reducing unhelpful narratives that could put siblings at an increased risk of eating difficulties. Family therapy may also be helpful in encouraging siblings to discuss their own difficulties and in assisting parents in managing these alongside supporting their child with AN.

A challenge in the delivery of family therapy for the treatment of AN is that it places responsibility on parents to manage the feeding of their unwell child and assumes that siblings are in a position to support this. This research highlights potential issues with this for siblings in that it may encourage them to take on responsibilities that feel overwhelming, to hide their own difficulties and may increase an unhealthy focus on food and body size. Family interventions may therefore need to carefully consider the nuances of family roles and relationships and the potential consequences of change to these throughout treatment. Consideration should be given to the involvement of siblings in structured eating plans and whether this may be detrimental to them. For siblings that develop eating difficulties, thought should be given to how to allow them sufficient space from unwell siblings to encourage recovery.

Many participants in this study highlighted a lack of awareness from schools or healthcare services of the impact that their sibling’s illness had on them. The burden of responsibility for their wellbeing fell mainly on their parents. This is problematic for children of parents that are already feeling overstretched and risks neglect for children of abusive parents. Although only two participants in this study highlighted parental abuse, their experiences demonstrate the increased risk of isolation that may face people in this situation and resources should be targeted at offering support to siblings of AN when abuse in the family has been identified.

### Further research

This research has identified some areas that may be associated with an increased risk of eating difficulties in siblings of people with AN. Further research is needed into the impact of particular risks, such as traumatic family events and the effects of people comparing their body size to that of a sibling with an eating disorder. Future studies may also consider how best to treat cases where more than one individual has AN concurrently. Additionally, investigation into the experiences of siblings who participate in family treatments for eating disorders would be beneficial.

Further research is needed into the impact of anti-obesity messaging within families and institutions (such as schools and health services) on eating disorder pathology, particularly in siblings and peers of people with AN, given the possible contagion in these groups [33, 34].

Finally, research into the efficacy of preventative interventions for siblings of people with AN would be valuable. Some evidence suggests that a support group for siblings of people with AN may be helpful but is it unclear whether this is protective against the development of eating disorder pathology [64].

## Conclusion

Siblings of people with AN have an increased genetic and environmental risk for disordered eating. This research suggests that the experience of witnessing a sibling with AN can impact on family structures and bring up difficult emotions and may affect wellbeing and restrictive eating. Additionally, an increased focus on food and body size at home, comparisons between siblings and emotionally charged mealtimes could influence eating patterns. Barriers to seeking support such as not wanting to burden family members and not seeing their difficulties as deserving of intervention were present. However, a desire to avoid the negative consequences of AN and a wish not to become as unwell as their sibling provided motivation for participants to eat healthily. Increased offers of support for siblings of people with AN to help identify those at risk of developing an eating disorder and to offer preventative treatments or early intervention would be beneficial.

## Supplementary Information

**Additional file 1.** **Appendix A.** Semi-structured Interview. Questions

## Data Availability

The datasets generated and analysed during the current study are confidential due to the need to protect the privacy of participants.

## Abbreviation

AN: Anorexia nervosa
