# Supplementary material for: Experiences of eating difficulties in siblings of people with anorexia nervosa: a reflexive thematic analysis
Source: J Eat Disord. 2022 Aug 20;10:123. doi: 10.1186/s40337-022-00646-2 (PMC9392277; doi:10.1186/s40337-022-00646-2)
Supplement: Supplementary file 1 — Additional file 1. Appendix A. Semi-structured Interview. Questions [file 40337_2022_646_MOESM1_ESM.docx]

# Appendix A

**Semi-structured Interview Questions**

Thank you for agreeing to participate in this research, as you are aware participation is voluntary and you may choose to end the interview or to skip a question at any time. This research is interested in your experiences as someone who has witnessed a sibling with Anorexia and who has experienced eating difficulties yourself. The questions I will ask are designed to find out more about your experiences, some of them may be difficult to answer or sensitive and you may ask to pause at any time. At times I may ask for further information on a question or for a bit more detail, this is to make sure that I have understood your experience and can accurately summarise this for the research. There are no correct or expected answers, you are the expert on your experiences so please share as much as you feel comfortable to.

**General Experiences**

1. Please could you give me an overview of your experiences of having eating difficulties
2. What were your experiences of living with a sibling with Anorexia nervosa/an eating disorder?
3. What support would you have liked when your sibling was first experiencing eating difficulties?
4. What, if anything, do you think could have been done to prevent you from developing eating difficulties?
5. Do you recall any significant events from your childhood that may have impacted on your eating difficulties?

**Family Functionin**g

1. What were your family relationships like before your sibling became unwell?
2. How did your sibling’s anorexia nervosa affect your relationship with your family members?
3. How did your relationship with your sibling change after they became unwell?
4. How have your own eating difficulties affected your family relationships?
5. Did you have any other significant relationships that helped or hindered your wellbeing?
6. What was the effect of your sibling’s difficulties on your own body image?

**Emotional Regulation/Experience of Trauma**

1. What emotions did you experience in relation to your sibling’s illness?
2. How did you manage these emotions?
3. What support would you have liked to help you manage emotions and difficulties following your sibling’s illness?
4. Did your sibling’s health have any influence on your illness or recovery?
5. What strengths do you have that have helped you to overcome these difficulties?
